# Integrated Transcriptomic and Metabolomic Analysis of the Mechanism of Foliar Application of Hormone-Type Growth Regulator in the Improvement of Grape (*Vitis vinifera* L.) Coloration in Saline-Alkaline Soil

**DOI:** 10.3390/plants11162115

**Published:** 2022-08-15

**Authors:** Doudou Chang, Huaijin Liu, Mengjie An, Dashuang Hong, Hua Fan, Kaiyong Wang, Zhiqiang Li

**Affiliations:** 1 Agricultural College, Shihezi University, Shihezi 832003, China; 2 Grape Research Institute, Shihezi 832003, China

**Keywords:** grape, quality, regulator, transcription factors, metabolite

## Abstract

(1) Background: To solve the problems of incomplete coloration and quality decline caused by unreasonable use of regulators in grapes, this study clarified the differences in the effects of a hormone-type growth regulator (AUT) and two commercial regulators on grape coloration and quality through field experiments. (2) Methods: The color indexes (brightness (L*), red/green color difference (a*), yellow/blue color difference (b*), and color index for red grapes (CIRG)) of grape fruit were measured using a CR-400 handheld color difference meter. The titratable acid content, total phenol content, and total sugar content were measured using anthrone colorimetry, folinol colorimetry, and NaOH titration, respectively, and the chalcone isomerase activity, phenylalanine ammoniase activity, dihydroflavol reductase activity, and anthocyanin content were measured using a UV spectrophotometer. (3) Results: The a*, total sugar and total phenol contents, and chalcone isomerase (CHI) and phenylalanine ammoniase (PAL) activities of grape fruit in the AUT treatment significantly increased, while the titratable acid content significantly decreased, compared to those in the CK treatment. The expressions of the differentially expressed genes (DEGs) *trpB* and *argJ* in AUT treatment were significantly up-regulated. The expressions of the differentially expressed metabolites (DEMs) phenylalanine and 4-oxoproline were significantly up-regulated, while those of 3,4-dihydroxybenzaldehyde and N-acetyl glutamate were significantly down-regulated. The CIRG significantly increased by 36.4% compared to that in the CK, indicating improved fruit coloration. (4) Conclusion: The AUT could shorten the color conversion period of grape fruit and improve the coloration, taste, and tolerance to saline and alkaline stresses.

## 1. Introduction

In arid areas, there is much less precipitation than evaporation. Long-term use of high-salinity groundwater for irrigation can lead to accumulation of salts in the surface soil [1]. This high-salinity and low-moisture soil can reduce the color indexes of fruit, increase the accumulation of total acids and total phenols [2], and reduce the size and weight of fruit. Previous studies have shown that salt stress may lead to decreased anthocyanin content and chalcone isomerase activity (CHI), prolonged coloration time, uneven fruit color, and inconsistent maturity, which ultimately lead to decreased grape fruit commodity value [3,4]. However, Chen et al. [5] found that foliar application of growth regulators could improve berry coloration, but different regulators had different effects on fruit coloration and taste. Therefore, the development of appropriate regulators is of great significance for grape production in arid areas.

Foliar application of growth regulators can enhance crop tolerance to environmental stresses and regulate fruit coloration, sugar content, and taste [6,7]. Growth regulators such as abscisic acids (ABA) and methyl jasmonate (*MeJA*) can obviously increase the accumulation of anthocyanins and improve fruit color [8]. Li [9] found that the application of ABA could regulate the expression of genes related to carotenoid metabolism, such as *PSY*, to improve fruit coloration. Chen et al. [5] found that the application of *MeJA* could up-regulate the expression of genes related to flavonoid biosynthesis, such as *CHSs*, *CHIs*, and *hct*. Garde-Cerdán et al. [10] found that the application of ABA could increase the activities of *CHI* and *PAL*, reduce acid content, and enhance fruit taste. Conde et al. [11] found that application of kaolin could promote the expression of genes such as *VvPAL1*, *VvC4H1*, *VvSTSs*, *VvFLS1*, *VvDFR*, and *VvUFGT* and significantly up-regulate the flavonoid (biosynthesis of flavonols and anthocyanins) and stilbenoid synthetic pathways, thereby promoting the biosynthesis of phenols and anthocyanins. Shi [12] found that the application of Fe-EDDHA could improve grape fruit taste by increasing the flavonol content. Li [13] found that the application of fulvic acid antitranspirant could promote the accumulation of hexyl acetate and linalool in fruit and improve fruit taste. Therefore, different growth regulators have different effects on fruit quality. However, the regulators cannot regulate fruit coloration and taste at the same time.

Although some growth regulators can improve the coloration or taste of grapes in arid areas, their monofunctionality cannot meet requirements in practice. Therefore, this study determined the effects of the foliar application of a self-developed hormone-type growth regulator (AUT) and two commercial regulators on grape fruit coloration and other qualities in arid areas. The aims were to: (1) clarify the differences in the effects of AUT and commercial regulators on the physiological and biochemical indexes of grape fruit, such as coloration and sugar content; and (2) determine the key pathways and metabolic pathways of the AUT’s regulation of grape coloration and other qualities. This study proposes a multifunctional growth regulator that can greatly improve the coloration, taste, and tolerance to environmental stresses of grape fruit in arid and semi-arid areas.

## 2. Materials and Methods

The experiment was conducted in Shihezi city, Xinjiang province, China (44°33′45.3″ N, 86°02′7.82″ E), from early May to the end of August 2019. The study site has a temperate continental climate with annual sunshine duration of 2, 721-2, 818 h, annual average rainfall of 200 mm, annual average evaporation of 1 and 250 mm, and a frost-free period of 147~191 days. The physicochemical properties of the topsoil (0–20 cm) were as follows: the pH was 8.35, the electric conductance (EC) (1:5) was 3.3 dS·m^−1^, the alkali hydrolyzable nitrogen content was 45.30 mg/kg, the available phosphorus content was 18.30 mg/kg, the available potassium content was 450 mg/kg, and the organic matter content was 13.80 g/kg.

### 2.1. Experimental Design

Five-year-old grape plants (“Red Earth”, a late maturing grape variety) with similar sizes and no diseases or insect pests were selected for the experiment. The plant spacing and row spacing were 0.8 m and 2.4 m, respectively. Fertilization followed the local practice (N: 170 kg/hm^2^, P_2_O_5_: 160 kg/hm^2^, and K_2_O: 220 kg/hm^2^). Drip irrigation was employed, with an irrigation volume of 11,100 m^3^/hm^2^. Ten plants with similar sizes and no plant diseases or insect pests were selected from each treatment on a sunny day on 1 July. On 10 July, deionized water (CK), nutrition-type growth regulator (YS), fulvic acid-type growth regulator (ZS), and self-developed hormone-type growth regulator (AUT) (patent number: 2020200435) were sprayed on the backs of leaves separately (Table 1). Each treatment had three replicates.

To determine which components and functional groups of the growth regulators affected the coloration and quality of the grape fruit, the regulators were measured with a Fourier-transform infrared spectroscope (Thermo Scientific Nicolet iS20). Samples were powdered. Then, 1~2 mg powder and 200 mg pure KBr were ground evenly. After that, the mixture was transferred to a mold, pressed into a transparent sheet on a hydraulic press, and tested with an infrared spectrometer (Thermo Scientific Nicolet iS20). The wave-number range was 4000~400 cm^−1^, the number of scans was 32, and the resolution was 4 cm^−1^ [14].

Sampling was performed 7, 14, 21, and 28 days after spraying water (CK) and regulators (YS, ZS, and AUT), until the fruit color no longer changed significantly. Grape fruit were selected from the upper, middle, and lower parts of different bunches, and a total of sixty fruit with uniform growth were selected. Thirty fruit were used for the determination of the single fruit weight (SW), vertical (VD) and horizontal (HD) diameter (VHD), peel hardness (PD), and the total sugar, total phenol, and titratable acid contents. The average values were calculated. The remaining thirty grape fruit were peeled off immediately in a clean environment, mixed evenly, and stored in liquid nitrogen and then in a −80 °C refrigerator for the determination of color indexes and transcriptomic and metabolomic analyses.

### 2.2. Determination of the Quality of Grape Fruit

The color indexes of the grape fruit were measured using a CR-400 color difference meter (Konica Minolta, Tokyo, Japan). Ten grapes with uniform growth were selected from each treatment to measure the color indexes, including brightness (L*), red/green color difference (a*), and yellow/blue color difference (b*), at the equator of each fruit. Each index was determined three times. L* represents the color brightness, which is in the range of 1~100. The value range of a* is [−60, +60] (a positive value means red and a negative value means green). The value range of b* is [−60, +60] (a positive value means yellow and a negative value means blue). The greater the absolute value, the darker the color. Then, the color index for the red grapes (CIRG) was calculated. If the CIRG < 2, the color is yellow green; if 2 < CIRG < 4, the color is pink; if 4 < CIRG < 6, the color is deep red; if CIRG > 6, the color is blue black.

(1) $$CIRG=\left(180–h{}^{\circ}\right)\;/\;\left(L^{*}+\;C^{*}\right)$$

(2) $$h{}^{\circ}=Arctan\;\left(\frac{b^{*}}{a^{*}}\right)$$

The PD was determined using a hand-held refractometer and a digital tension meter (HG2000, Grows, Shanghai, China). The SW from thirty fruit was determined using an electronic balance (PRACTUM124-1CN, Sartorius, Germany), and the VD and HD were determined using a vernier caliper (160-131, Mitutoyo, Kamala, Japan).

### 2.3. Determination of Total Sugar, Total Phenol and Total Acid Contents

The total sugar content was determined with the anthrone colorimetric method [15]. The total phenol content was determined with the Folin colorimetric method [16]. The titratable acid content was determined with the NaOH titration method. Grape juice (5 mL) for each treatment was mixed with 15 mL of distilled water, and the mixture was titrated with 0.1% NaOH using 1% phenolphthalein as indicator [17].

### 2.4. Determination of Physiological Indexes in Grape Skins

The extract was prepared with reference to the method described by Lister [17], and the CHI activity and PAL activity were determined colorimetrically at 381 nm and 290 nm, respectively. The crude enzyme solution of DFR was prepared with reference to the method described by Lister [18]. Enzyme solution (600 μL) was mixed with 400 μL extraction solution, and then subjected to a water bath at 36 °C for 1 h. After that, ethylacetate was added to terminate the reaction, and the supernatant was placed in an evaporation dish until complete evaporation following cooling and stratification. Then, n-butanol HCl solution was added. The mixture was subjected to a water bath at 100 °C for 20 min, and the absorbance was measured at 550 nm using a UV-Vis spectrophotometer (I3, Jinan Haineng Instrument Co., Ltd., Jinan, China). Anthocyanin content was determined using the 2% hydrochloric acid methanol immersion method [19], and a UV-Vis spectrophotometer (I3, Jinan Haineng Instrument Co., Ltd., Jinan, China) was used for the determination at 530 nm and 700 nm. The calculation formulas were as follows:

(3) $$\mathrm{Anthocyanin}\;\mathrm{content}\;\left(\mathrm{mg}/g\;\mathrm{FW}\right)=\left(D\times S\times \mathrm{PF}\times 1000\right)/26,900$$

(4) $$D=\left(A520-\;A700\right)\mathrm{PH}1.0-\left(A520-\;A700\right)*\mathrm{PH}4.5$$

where S is 449.2 and PF is the dilution factor of the extract.

Through the determination and comparison of the appearance characteristics and physiological indexes of the grape fruit with different treatments, it was found that different regulators had different effects on grape coloration, and the AUT had the most significant effect on grape coloration. Therefore, the AUT and CK treatments were selected for the transcriptomic and metabolomic analyses to reveal the ways that AUT regulated grape coloration and other quality indexes at the molecular level.

### 2.5. Transcriptome Analysis

Grape peel was collected and quickly cooled with liquid nitrogen. RLT buffer (0.5 mL) and mercaptoethanol were transferred into a 1.5 mL centrifuge tube. The sample (0.1 g) was ground with liquid nitrogen, mixed with the extract solution, bathed in water at 56 °C for 2 min, and centrifuged at 12,000 rpm at 23 °C for 15 s. The filtrate was then transferred into a new 1.5 mL centrifuge tube, and one-half volume of absolute ethanol was added. The mixture was centrifuged at 12,000 rpm at 23 °C for 15 s. After discarding the filtrate, 700 μL RW1 was added, followed by centrifugation at 12,000 rpm at 23 °C for 15 s. The column was placed on a new 2 mL tube. Then, 500 μL RPE was added, and the mixture was centrifuged at 12,000 rpm at 23 °C for 15 s. After discarding the filtrate, 500 μL RPE was added, followed by a centrifugation at 12,000 rpm at 23 °C for 2 min. Then, ddH_2_O (20 μL) pre-treated with DEPC was added and centrifuged at 12,000 rpm at 23 °C for 1 min after 15 min. After that, 40 μL and 20 μL ddH_2_O pre-treated with DEPC were added and centrifuged at 12,000 rpm at 23 °C for 1 min after 15 min. All collected RNAs were mixed, and 2 μL electrophoresis was used to detect the purity and quality. Samples were stored at −20 °C for a short time and then at −80 °C. Simultaneously, the same amounts of samples were mixed to prepare the QC samples, which were re-dissolved using 50 μL isopropanol/methanol/water (*V*/*V*/*V* = 1:1:2), and the supernatant was centrifuged for transcriptomic analysis [20].

### 2.6. Metabolomic Analysis

Grape peel was collected, cooled with liquid nitrogen, and stored in a refrigerator at −80 °C. After grinding with liquid nitrogen, 100 mg of the sample was weighed and transferred into a 1.5 mL EP tube. Then, 400 μL precooled methanol/water solution (*V*/*V* = 3:1) was added, vortexed, stored at 4 °C overnight, and centrifuged at 13,000 rpm at 4 °C for 15 min. The supernatant was passed through a 0.22 μm filter membrane, dried with nitrogen, and stored at −80 °C for use. Simultaneously, the same amounts of samples were mixed to prepare the QC samples. QC samples (100 mg) were mixed with 400 μL precooling methanol/water (*V*/*V* = 3:1), vortexed, stored at 4 °C for 2 h, and centrifuged at 13,000 rpm at 4 °C for 15 min. The supernatant was passed through a 0.22 μm filter membrane, dried with nitrogen, and stored at −80 °C for use. Before LC-MS mass spectrometry, samples were re-dissolved using 50 μL isopropanol/methanol/water (*V*/*V*/*V* = 1:1:2), and the supernatant was centrifuged for metabolomic analysis [21].

### 2.7. Statistical Analysis

The indexes were analyzed with one-way ANOVA using SPSS 19.0 software (IBM, Armonk, NY, USA) (Duncan test, *p* < 0.05), and the plotting was performed using Origin 18.0 (Origin Laboratories, Ltd., Northampton, MA, USA). Fastp, a FASTQ data pre-processing tool, was used for quality control of the original sequencing data [22]. The reference genome of grape (GCA_000003745.3 12X/) and annotation files were obtained from the National Center for Biotechnology Information (NCBI) [23]. Deseq2 was used for differential expression analysis [24], and |log_2_foldchange| > 1 and *p* < 0.05 were used as the threshold. The KEGG annotations are a collection of HTML tables called BRITE tables [25], and the DEGs were enriched and analyzed using the clusterProfiler package in R software (version 3.2.3, http://www.r-project.org accessed on 7 June 2021) [26]. DEMs were selected with a threshold of VIP > 1 and 0.05 < *p* < 0.1.

## 3. Results

### 3.1. Fourier-Transform Infrared (FTIR) Spectroscopy Analysis of Different Regulators

**Figure 1:**
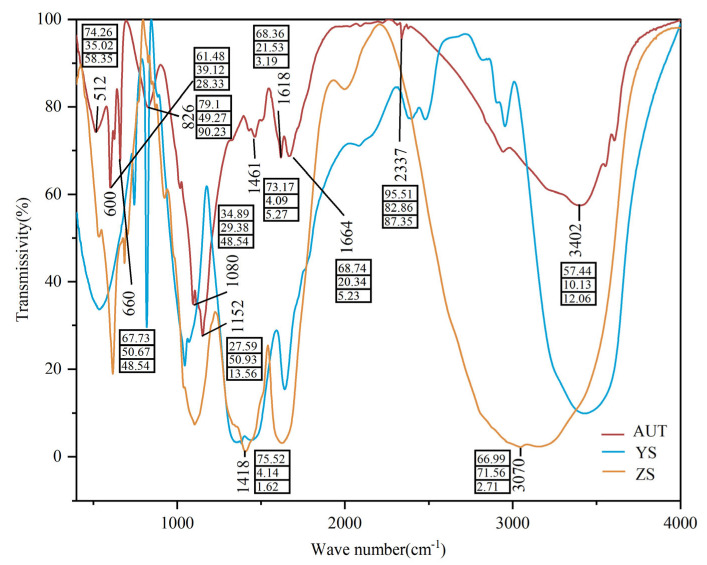
FTIR spectra of growth regulators. AUT, YS, and ZS represent a self-developed hormone-type growth regulator, nutrition-type growth regulator, and fulvic acid-type growth regulator (ZS), respectively. The data represent relative intensities of major absorption peaks of FTIR spectra of different treatments (semi-quantitative).

The results of the FTIR showed that the peaks at 1461 cm^−1^ and 1618 cm^−1^ were attributed to the variable-angle vibrations of CH_3_ and H_2_O in the regulators, respectively (Figure 1). The peak at 3402 cm^−1^ was attributed to the stretching vibration of NH_2_ of aromatic secondary amines. The peaks at 1094 cm^−1^ and 1152 cm^−1^ were attributed to the rocking vibration of CH_3_ and the C-OH stretching vibration of phenols, respectively. The peaks at 600 cm^−1^, 660 cm^−1^, and 860 cm^−1^ were attributed to the vibration of inorganic phosphate, the rocking vibration of H_2_O, and the vibration of CO_3_^2−^, respectively. The peak at 1418 cm^−1^ was attributed to the stretching vibration of COO. Compared with YS and ZS, AUT had higher absorbances at 3402 cm^−1^, 1664 cm^−1^, 1461 cm^−1^, 1418 cm^−1^, 660 cm^−1^, 600 cm^−1^, and 512 cm^−1^. YS had the highest absorbance at 3070 cm^−1^, and ZS had the highest absorbances at 826 cm^−1^ and 1094 cm^−1^.

### 3.2. Effects of Growth Regulators on the Appearance Characteristics of Grape Fruit

**Figure 2:**
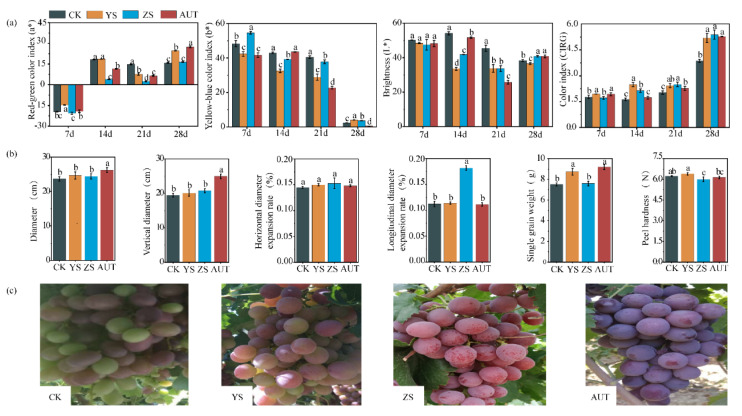
Changes in grape quality after application of growth regulators. The four pictures from left to right show the changes in the red/green color difference, yellow/blue color difference, brightness, and CIRG of grape fruit in the four treatments (**a**). The six pictures from left to right show the horizontal diameter, vertical diameter, horizontal diameter increase rate, vertical diameter increase rate, single fruit weight, and peel hardness of grape fruit in the four treatments on day 28 (**b**). The appearance of the grape fruit in the four treatments on day 28 (**c**). Different lowercase letters indicate significant differences between treatments on the same day (*p* < 0.05), and the same notation is used below.

Growth regulators obviously affected the appearance of grape. The grape fruit in the AUT treatment had the optimal a*, L*, CIRG, VHD, and SW values. The a* value gradually changed from a negative value to a positive one. The a* values in the YS and AUT treatments were 55.3% and 72.2% higher than those in the CK treatment on day 28, respectively (*p* < 0.05). The b* value in each treatment decreased obviously after 21 days, and the b* value in the AUT treatment was 13.1% lower than that in the CK (*p* < 0.05). The L* value in each treatment decreased obviously after 7 days. The L* value in the ZS and AUT treatments were 6.27% and 6.33% higher than that in the CK treatment on day 28, respectively (*p* < 0.05). The CIRG values in the three regulator treatments were all higher than that in the CK at all stages and reached the peak on day 28. The CIRG values in the YS, ZS, and AUT treatments were 34.2%, 39.3%, and 36.4% higher than that in the CK treatment, respectively (*p* < 0.05) (Figure 2a). On day 28, the VD, HD, and SW values in the AUT treatment were 27.8%, 10.6%, and 22.4% higher than those in the CK treatment, respectively (*p* < 0.05), while there was no difference in the ZS value (*p* < 0.05) (Figure 2b). Furthermore, the fruit colors in the three growth regulator treatments were all better than that in the CK. Among them, the fruit color in the AUT treatment was the best, followed by that in the ZS treatment (Figure 2c).

### 3.3. Effects of Growth Regulators on Biochemical Characteristics of Grape Pulp

**Figure 3:**
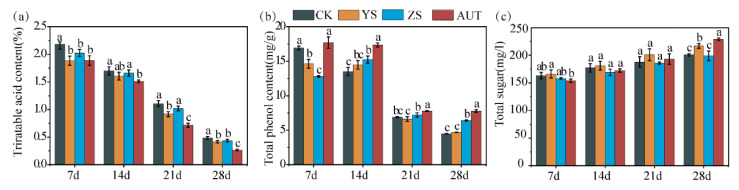
Changes in biochemical characteristics (titratable acid content (**a**), total phenol content (**b**), and total sugar content (**c**)) of grape fruit in different treatments. Values marked with different letters differ significantly from each other (*p* < 0.05).

The growth regulators, especially AUT, obviously improved the biochemical characteristics of grape pulp. The grape fruit in the AUT treatment had the lowest titratable acid content and the highest total phenol and total sugar contents. The titratable acid contents for the grape fruit in the YS, ZS, and AUT treatments were 17.4%, 11.4%, and 81.5% lower than that in the CK treatment, respectively, on day 28 (*p* < 0.05) (Figure 3a). The total phenol content of the grape fruit in the AUT treatment was the maximum across the whole color conversion period. The total phenol contents in the ZS and AUT treatments were 43.0% and 73.0% higher than that in the CK treatment, respectively, on day 28 (*p* < 0.05) (Figure 3b). The total sugar content gradually increased with time. There was no difference in the total sugar contents among the treatments on day 21, while the total sugar contents in the YS and AUT treatments were 8.0% and 13.9% higher than that in the CK treatment, respectively, on day 28 (*p* < 0.05) (Figure 3c).

### 3.4. Effects of Growth Regulators on the Activities of Enzymes Related to Coloration of Grape Fruit

**Figure 4:**
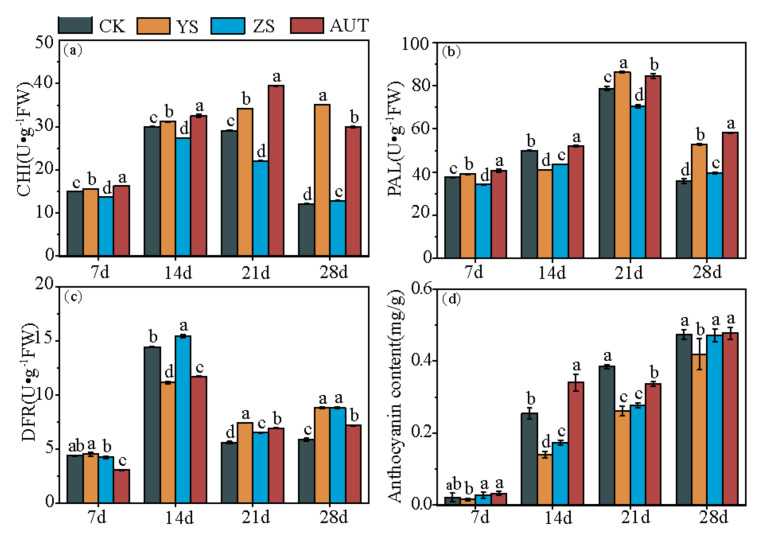
Changes in the anthocyanin content and the activities of enzymes related to grape coloration (CHI (**a**), phenylalanine ammoniase activity (**b**), dihydroflavol reductase activity (**c**), and anthocyanin content (**d**)). Values marked with different letters differ significantly from each other (*p* < 0.05).

Growth regulators had obvious effects on the enzyme activities of the grape fruit during the color conversion period. The enzyme activities in the regulator treatments (especially the YS and AUT treatments) were all higher than those in the CK treatment. There was an obvious difference in the CHI activity between treatments. The CHI activities in the YS and AUT treatments were 189.0% and 146.0% higher than that in the CK treatment, respectively, on day 28 (*p* < 0.05), while the CHI activity in the ZS treatment was lower than that in the CK treatment (*p* < 0.05) (Figure 4a). The PAL activity of fruit in each treatment reached the peak on day 21. The PAL activities in the YS and AUT treatments were 47.3% and 62.5% higher than that in the CK, respectively, on day 28 (*p* < 0.05), and there was no difference between the ZS and CK treatments (Figure 4b). The DFR activity of the fruit in each treatment reached the peak on day 14. The DFR activities of the fruit in the YS, ZS, and AUT treatments were 50.4%, 50.8%, and 22.5% higher than that in the CK treatment, respectively, on day 28 (*p* < 0.05) (Figure 4c). The anthocyanin content of fruit in each treatment increased gradually and reached a peak on day 28. There was no difference in anthocyanin contents between the ZS and AUT treatments and the CK treatment, but the anthocyanin content in the AUT treatment was higher than that in the CK treatment on day 14 (Figure 4d).

### 3.5. Effects of Growth Regulators on Transcriptomics and Metabolomics of Grape

**Figure 5:**
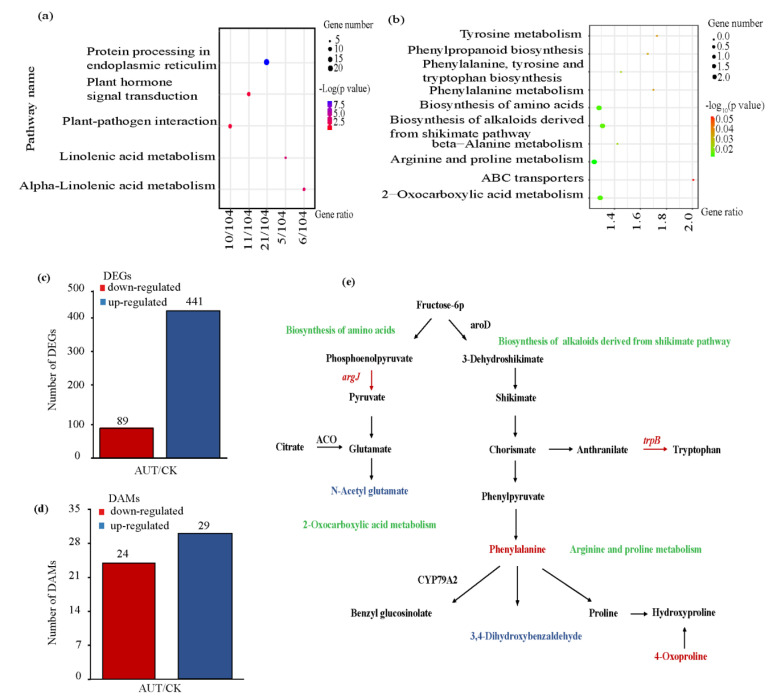
The KEGG pathways enriched by differentially expressed metabolites (DEMs) in grape peel (**a**). The KEGG pathways enriched by differentially expressed genes (DEGs) in grape peel (**b**). The number of DEGs in grape peel (**c**). The number of DEMs in grape peel (**d**). Simplified overview of the pathways leading to the changes in color and quality in grape fruit (**e**). Green represents the pathways involving coloration and quality, blue represents up-regulated metabolites, and red represents down-regulated transcription factors and metabolites.

After foliar application of AUT, 530 DEGs were identified, including 89 down−regulated genes and 441 up−regulated genes compared to the CK treatment (Figure 5c). A total of 10,621 genes and 222 DEGs were annotated in GO database. KEGG enrichment analysis showed that a total of 4214 genes and 104 DEGs were annotated, involving 114 KEGG pathways. The top five enriched pathways are shown in Figure 5a. Most DEGs were enriched in the pathways related to protein processing in the endoplasmic reticulum, plant hormone signal transduction, and plant–pathogen interaction, and a few were enriched in the pathways related to linoleic acid metabolism and alpha-linolenic acid metabolism.

There were 53 DEMs (VIP > 1 and *p* < 0.05) in the AUT treatment, among which the expressions of 29 metabolites were up-regulated and those of 24 metabolites were down−regulated compared with the CK treatment (Figure 5d). The DEMs, such as phenylalanine, N−acetyl glutamate, 3,4−dihydroxybenzaldehyde, and 4−oxoproline, were related to grape coloration and quality. The KEGG enrichment analysis showed that, among the top ten pathways, the pathways for arginine and proline metabolism and biosynthesis of amino acids were related to fruit coloration, and 2−oxocarboxylic acid metabolism and biosynthesis of alkaloids derived from the shikimate pathway were related to the accumulation and metabolism of phenolic substances in fruit (Figure 5b).

The integrated transcriptomic and metabolomic analysis showed that the application of AUT induced the up−regulation of the expression of *trpB* in the biosynthesis of alkaloids derived from the shikimate pathway and of phenylalanine and 4−oxoproline in the arginine and proline metabolism pathway, and the down-regulation of 3,4− dihydroxybenzaldehyde in the arginine and proline metabolism pathway (*p* < 0.05). In the biosynthesis of amino acids pathway, the application of AUT stimulated the up−regulation of *argJ*, leading to the up−regulation of N−acetyl glutamate (*p* < 0.05) (Figure 5e).

### 3.6. Network Analysis of Transcription Factors and Metabolites Related to Grape Quality

**Figure 6:**
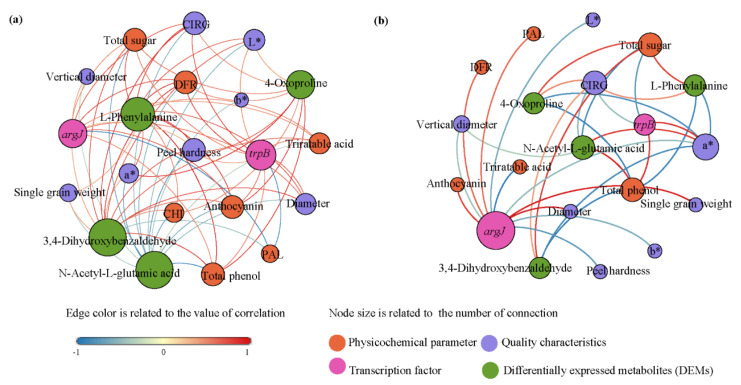
Co-occurrence network analysis of physicochemical indexes, quality characteristics, and transcription factor, and differentially expressed metabolites in response to CK (**a**) and AUT (**b**).

Foliar application of AUT changed the correlations of color indexes and physiological indexes in the co−occurrence network (Figure 6). In the CK, the DEMs, including L−phenylalanine, 3,4−dihydroxybenzaldehyde, and 4−oxoproline, were negatively correlated with color indexes (a*, b*, L*, and CIRG), physiological indexes (total sugar, total phenol, and titratable acid contents), and enzyme activities (PAL activity, CHI activity, and DFR activity). N−acetyl−l−glutamic acid was positively correlated with color and physiological indexes. *argJ* was positively correlated with PAL activity and negatively correlated with physiological indexes. *trpB* was negatively correlated with L* and CIRG and negatively correlated with physiological indexes. The DEMs, including N−acetyl glutamate, L−phenylalanine, and 4−oxoproline, were the main regulatory factors and had a great influence on the color indexes of grape. In the AUT treatment, *trpB* was positively correlated with the total sugar content and CIRG and negatively correlated with a* and the total phenol content. L−phenylalanine and a* were positively correlated with the total phenol content and negatively correlated with the total sugar content. N−acetyl glutamate and *trpB* were negatively correlated with a* and the total phenol content and positively correlated with CIRG, the total sugar content, and the VD. 4−Oxoproline was positively correlated with a* and the total phenol content and negatively correlated with CIRG and the total sugar content.

## 4. Discussion

In this study, foliar application of AUT improved the activity of coloration-related enzymes and the abundance of *trpB* and accelerated the coloration of grape fruit in saline-alkali soil. Furthermore, AUT application advanced the completion of coloration by 7 days, and the fruit color quality was also better than that in the CK treatment. The reasons are as follows. On the one hand, it was found that, after spraying AUT, fruit color changed with the ripening of the grape fruit from green to red, and the b* value reached the minimum value on day 21. This indicates that AUT can shorten the color conversion period (from yellow to blue). This is consistent with the study results described by Ma et al. [27]. They obtained similar result after spraying 1-naphthylacetic acid (NAA) and ABA on citrus foliage. On the other hand, it was found that AUT application significantly increased the a*, b*, and CIRG values and reduced the L* value of the grape fruit. However, Wang et al. [28] found that application of exogenous 5-aminolevulinic acid significantly reduced the a*, b*, and L* values in tomato. Therefore, there are differences in the effects of different regulators on fruit coloration. Fruit coloration is jointly affected by a variety of pigments, and anthocyanins are commonly used to reflect the degree of fruit coloration [29]. Therefore, in this study, to explore the physiological mechanism of AUT’s regulation of grape fruit coloration, the activities of coloration-related enzymes and the of anthocyanin content were measured. It was found that the activity of PAL, an initiating enzyme for anthocyanin synthesis [30], was increased after spraying AUT, and the fruit color was deepened. CHI [31] and DFR [32] are involved in the synthesis of anthocyanins. Our study found that the activity of CHI was decreased after spraying AUT, and there was no significant difference for DFR. However, the accumulation of anthocyanins was only affected by a complete lack of CHI [31]. Thus, AUT application could not inhibit fruit coloration. Next, to reveal the molecular mechanism of AUT’s regulation of grape fruit coloration, transcriptomic and metabolomic analyses were carried out. It was found that *trpB* was significantly positively correlated with a*. Therefore, the up-regulation of *trpB* after spraying AUT directly promoted fruit coloration. The up-regulation of *argJ* detected in this study could increase the activities of PAL and DFR, which is beneficial to the the accumulation of anthocyanins [33]. Furthermore, it was also found that AUT application up-regulated the expression of amino acid metabolites, especially the expression of phenylalanine. The higher the phenylalanine content is, the redder the grape peel [34] (Figure 6). Therefore, the foliar application of AUT can significantly improve the color quality of grape fruit.

Our study results showed that, after spraying AUT, the soluble sugar and total phenol contents were increased in grape fruit, while the titratable acid content was reduced. Therefore, AUT application could improve grape fruit taste. This may be due to the up-regulation of *argJ* after spraying AUT. A study has shown that up-regulation of *argJ* can lead to increased accumulation of total phenols and reduced titratable acid content [35]. Furthermore, it was also found that the expression of phenylalanine was up-regulated, while that of 4-oxoproline was down-regulated, after spraying AUT. Phenylalanine has a strong correlation with phenol biosynthesis in crops [36]. However, a previous study [37] found that exogenous application of S-ABA increased the anthocyanin, delphinidin-3-glucoside, and cyanidin-3-glucoside contents, as well as the expression of the biosynthetic genes CHI, F3H, and DFR, in grape fruit peel. This shows that the application of regulators can change different transcription factors and metabolites related to fruit quality. The reason may be that different regulators have different functional groups with different characteristics [38]. Therefore, the functional groups of AUT affecting grape fruit coloration were analyzed using FTIR in this study. After spraying AUT, the accumulation of phenols in grape fruit was increased. This may have been due to the fact that brassinolide can penetrate the grape cell membrane and enhance the stretching vibration of C-OH. Guillermo et al. [39] showed that C-OH is closely related to the synthesis of alcohols and phenols in fruit. Therefore, AUT application can ultimately improve the accumulation of phenols [40,41,42]. Furthermore, it was also found that the absorbance at the region of the stretching vibration of the COO amino acid group in AUT was higher than that in the other two regulators. This indicates that AUT can clearly promote the accumulation of phenols and organic acids in grape fruit [38]. The metabolomic analysis results further explained the changes in grape fruit taste. Our results showed that AUT application obviously promoted the accumulation of DEMs in grape peel. The DEM N-acetyl glutamate was negatively correlated with total sugar (Figure 6). The decreased relative accumulation of N-acetyl glutamate could suppress the biosynthesis of arginine [42] and promote greater sugar allocations in the syntheses of other amino acids, thus increasing the accumulation of soluble sugar and inhibiting the production of titratable acid [27]. Therefore, the components of the regulators were the main factors causing grape fruit taste changes.

## 5. Conclusions

The foliar application of AUT can improve grape fruit color and shorten the coloration period by improving the expression of the metabolite L-phenylalanine and the activities of CHI, PAL, and DFR through the up-regulation of the transcription factor *trpB* and the regulation of the biosynthesis of alkaloids derived from the shikimate pathway. Furthermore, it can also promote the synthesis of sugars and phenols in grape peel to enhance the taste of grape fruit through up-regulation of the expression of *argJ* to regulate the biosynthesis of amino acids and down-regulation of the expression of the metabolite N-acetyl glutamate.

## Figures and Tables

**Table 1:** Experimental design.

Treatment	Main Components	Application Rate
Control group (CK)	Water	400 mL/ha
Nutrition-type growth regulator (YS)	Ca ≥ 180 g/L, Mg ≥ 20 g/L, Zn ≥ 30 g/L, and B ≥ 5 g/L	400 mL/ha
Fulvic acid-type growth regulator (ZS)	Fulvic acid ≥ 1 million lu, amino acid ≥ 100 g/L, and Mg + Zn + B + Fe ≥ 20 g/L,	400 mL/ha
Hormone-type growth regulator (AUT)	N-propyl dihydrojasmonate ≥ 1 g/L, Brassinolide ≥ 0.5 g/L and Mg + Zn + B + Fe ≥ 20 g/L	400 mL/ha

## Data Availability

The data presented in this study are available in the article.
